# *Stenotrophomonas maltophilia* Complex: Genomic Characterization, Antimicrobial Resistance and First Report of *S. muris* from Oman

**DOI:** 10.3390/antibiotics15060600

**Published:** 2026-06-12

**Authors:** Amira ElBaradei, Atika Al-Bimani, Suad A. H. Al-Ubaidani, Amal Al-Hinai, Zainab J. Al-Lawati, Hafidha Al-Hattali

**Affiliations:** 1 Department of Microbiology and Immunology, College of Medicine and Health Sciences, Sultan Qaboos University, Muscat 123, Oman; a.elbaradei1@squ.edu.om (A.E.); bimani2012@squ.edu.om (A.A.-B.); amhinai@squ.edu.om (A.A.-H.); zallawati@squ.edu.om (Z.J.A.-L.); 2 Microbiology and Immunology Diagnostic Laboratory, Sultan Qaboos University Hospital, University Medical City, Muscat 123, Oman; suad89@squ.edu.om; 3 Department of Biomedical Science, College of Medicine and Health Sciences, Sultan Qaboos University, Muscat 123, Oman

**Keywords:** *Stenotrophomonas maltophilia*, *Stenotrophomonas muris*, *sul1*, *blaOXA-2*, antimicrobial resistance, cefiderocol, MGEs, MLST, virulence, *stmPr1*

## Abstract

**Introduction:** *Stenotrophomonas maltophilia* (*S. maltophilia*) has emerged as an important opportunistic pathogen. It is resistant to most available antibiotics due to its intrinsic resistance, leaving only some antibacterial agents as possible therapeutic options, which is further complicated by acquired mechanisms of antimicrobial resistance. This study aimed to provide a comprehensive genomic characterization of clinical *S. maltophilia* complex (Smc) isolates, focusing on molecular characterization of its resistance and virulence, since studies tackling this are scarce in Oman. **Methods:** This study is a prospective cross-sectional study, in which a total of 21 clinical isolates of Smc were collected from different clinical samples and further characterized using Whole Genome Sequencing. **Results:** Besides *S. maltophilia*, the isolates included *S. hibiscicola*, *S. pavanii*, and *S. muris* for the first time in Oman. All isolates were found to be susceptible to cefiderocol, levofloxacin, and minocycline. Sequence types (STs) were diverse among the isolates, with more than half of the isolates showing new STs with novel alleles. Additionally, *blaOXA-2*, *sul1*, and the recently described *aac(6′)-Iap* and *aph(9)-Ic* were detected among the isolates. Moreover, virulence-associated genes (*smf-1*, *pilT*, *pilQ*, *gpmA*, *rmlA*, *spgM*, *stmPr1*, *plcN*, *clpP*, and *katE*) were highly conserved across all isolates. Mobile genetic elements were detected in most of the isolates (76.20%). **Conclusions:** The collected isolates showed high ST diversity and showed no specific pattern in terms of antibiotic susceptibility and resistance genes. More studies are needed to establish relationships between the different members of the Smc and the different molecular resistome and virulome.

## 1. Introduction

*Stenotrophomonas maltophilia* (*S. maltophilia*) is an aerobic, glucose non-fermenting, motile Gram-negative bacillus, which was previously known as *Pseudomonas maltophilia* or *Xanthomonas maltophilia*. It is abundant in different environmental surroundings [1,2].

*S. maltophilia* was deemed a low-virulence bacterium; however, it has emerged as an important opportunistic nosocomial pathogen, especially among debilitated patients, because of its ability to survive in hospital settings. It ranks in the third place after *Pseudomonas aeruginosa* and *Acinetobacter baumannii* as the most isolated unusual non-fermenting Gram-negative bacteria [2,3,4]. It is implicated in different types of infections; however, it is often recovered from patients with chronic lung diseases. Also, it causes infections in different sites, including respiratory tract infections, urinary tract infections, catheter-associated bloodstream infections, meningitis, and endocarditis [2,5,6,7].

*S. maltophilia* is recognized as a clinically important opportunistic pathogen with substantial intrinsic antimicrobial resistance, which complicates treatment and limits available therapeutic options [8]. Low membrane permeability, multidrug resistance efflux pumps, and aminoglycoside-modifying enzymes are among the different intrinsic mechanisms of antimicrobial resistance in *S. maltophilia* [9].

β-lactamases are enzymes that hydrolyze β-lactams and render them inactive. Generally, Ambler classification is the most used classification system to categorize these enzymes, relying on their amino acid similarities. According to Ambler classification, β-lactamases are grouped into four distinct classes: A, B, C, and D. Class B includes the metallo-β-lactamases, in which Zn^2+^ is essential for their activity, while the rest of the classes do not possess Zn^2+^ in their active site; instead, they possess the amino acid “serine” [10,11,12]. A major component of its intrinsic β-lactam resistance is the production of two chromosomally encoded β-lactamases, L1 and L2 [13]. L1 is a metallo-β-lactamase, whereas L2 is a serine β-lactamase; together, they contribute to the broad β-lactam resistance profile commonly observed in *S. maltophilia* [14].

*S. maltophilia* carries different virulence factors as well as various virulence-associated putative factors. These factors contribute to many aspects of its ability to effectively colonize and persist on surfaces as well as its ability to form biofilms on different surfaces, which increases resistance to antimicrobial agents and host immune responses. These factors include structures responsible for adhesion, like fimbriae, type IV pili, and adhesins, in addition to the production of enzymes such as proteases and lipases, as well as quorum sensing and different secretion systems [7,15,16].

*S. maltophilia* complex (Smc) is a group of closely related species that has been recently expanded to include mainly *S. maltophilia*, *S. beteli*, *S. forensis*, *S. geniculata*, *S. hibiscicola*, *S. muris*, *S. pavanii*, *S. riyadhensis*, and *S. sepilia* [17]. Genomic studies have shown that the Smc is highly heterogeneous, comprising multiple phylogenetic lineages and closely related species-level clades, which complicates species-level identification and epidemiological interpretation [18,19]. In particular, 16S rRNA gene sequencing has limited discriminatory power for closely related *Stenotrophomonas* species, while routine identification methods such as Matrix-Assisted Laser Desorption Ionization–Time of Flight Mass Spectrometry (MALDI-TOF MS) may be influenced by the breadth and quality of reference databases [19]. In addition, virulome interpretation remains challenging because many predicted virulence-associated determinants are putative and are often inferred using broad, non-species-specific databases rather than a dedicated *S. maltophilia* virulence framework [7]. Collectively, these challenges underscore the value of whole-genome sequencing in resolving species-level identity, defining resistome and virulome profiles, and enhancing genomic surveillance of Smc isolates.

Despite the growing recognition of Smc as an important opportunistic pathogen, genomic data from Oman remain scarce. Therefore, this study aimed to provide a comprehensive genomic characterization of clinical Smc isolates from Oman, with particular emphasis on antimicrobial resistance determinants, virulence-associated genes, mobile genetic elements, and phylogenetic relatedness. These findings may contribute to a better understanding of the local genomic epidemiology of Smc and its potential implications for clinical microbiology, infection control, and antimicrobial resistance surveillance.

## 2. Results

In this study, a total of 21 consecutive clinical isolates of Smc were collected, sequenced, and deposited in the National Center for Biotechnology Information (NCBI) database. To prevent patient-repetition bias, this dataset was deduplicated to 20 unique isolates for downstream demographic and phenotypic susceptibility profiling. The demographic characteristics, hospital ward distributions, and different types of samples of the 20 isolates are shown in Table 1.

**Figure 1:**
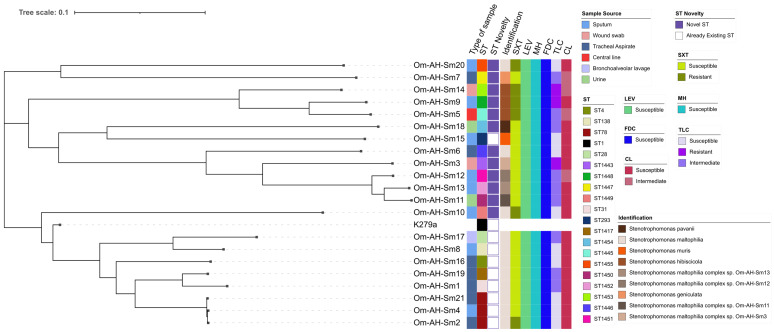
Phylogenetic tree of Smc clinical isolates (Om-AH-Sm1-21). Types of samples, ST, novel STs, identification, and antibiotic susceptibility patterns of the isolates are indicated with colored annotation tracks.

The clinical isolates of Smc that were collected from different clinical samples are shown in Figure 1. Most of the Smc clinical isolates (15/20, 75%) were obtained from respiratory tract infections, while only one isolate (1/20, 5%) was obtained from blood culture from a central-line catheter. Wound swabs and urine samples provided only two isolates (2/20, 10%) each.

All isolates in this study were susceptible to cefiderocol (FDC), levofloxacin (LEV), and minocycline (MH) (Figure 1). However, susceptibility was variable to chloramphenicol (CL), trimethoprim/sulfamethoxazole (SXT), and ticarcillin/clavulanic acid (TLC), with most of the isolates susceptible to CL followed by SXT and TLC. SXT-susceptible isolates were (14/20, 70%) of the isolates, and the rest were resistant to SXT. CL-susceptible isolates were (16/20, 80%) of the isolates, with the rest of the isolates being intermediate. Lastly, TLC showed the least susceptibility, with only (8/20, 40%) of the isolates being susceptible, (9/20, 45%) of isolates being intermediate, and the rest of the isolates (3/20, 15%) being resistant. The MIC values of CL and TLC are shown in Table 2.

The identification to the species level of the 21 isolates was confirmed by 16S rRNA sequencing used in WGS assembly quality control checks, and confirmed again using the KmerFinder 3.2 online tool. However, on submission to NCBI, ten of the isolates (10/21, 47.62%) were found to belong to species other than *S. maltophilia* using Average Nucleotide Identity (ANI) (Figure 1). Remarkably, one of the isolates (OM-AH-Sm15) was confirmed to be *S. muris*, and to the best of our knowledge, this is the first report of *S. muris* from a clinical sample in Oman. It is worth mentioning that all the identified species belonged to Smc. The phylogenetic relationship between the 21 isolates is shown in Figure 1.

The most detected STs were ST78 in three (14.29%) isolates. Ten isolates (47.62%) showed completely novel alleles, and the combination of the different alleles subsequently formed novel STs. Two other isolates (9.52%) had unique combinations of alleles that formed novel STs. These novel alleles and STs were submitted to pubMLST (Figure 1). Additionally, the different alleles and STs, including the novel ones, are shown in Table 3.

**Figure 2:**
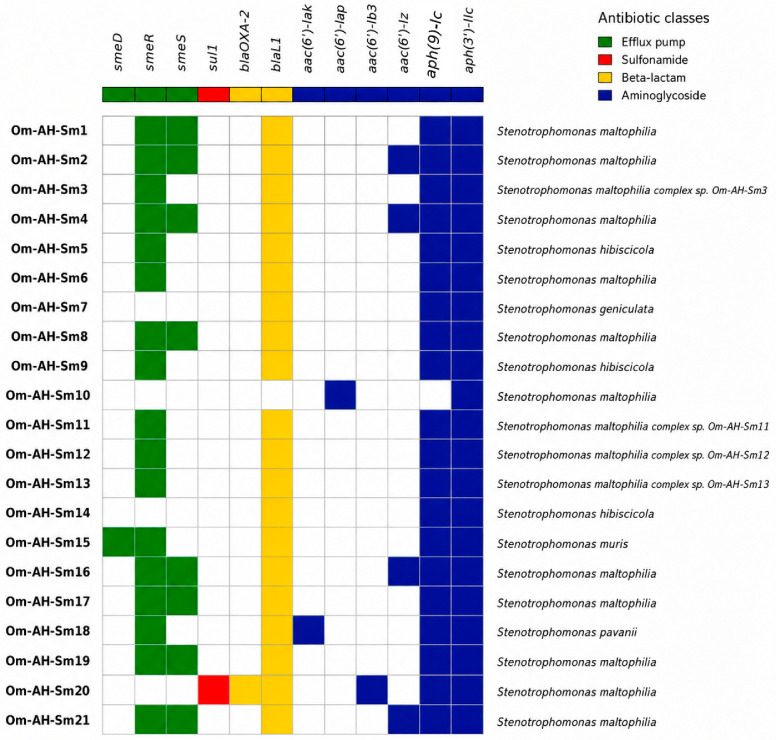
Heatmap showing the distribution of antibiotic resistance genes among Smc clinical isolates. The heatmap of antibiotic resistance genes was grouped into efflux pump-associated genes (*smeD*, *smeR*, and *smeS*), sulfonamide resistance gene (*sul1*), β-lactam resistance genes (*blaOXA-2* and *blaL1*), and aminoglycoside resistance genes (*aac(6′)-Iak*, *aac(6′)-Iap*, *aac(6′)-Ib3*, *aac(6′)-Iz*, *aph(9)-Ic*, and *aph(3′)-IIc*).

The distribution of antibiotic resistance genes among Smc in clinical isolates was identified (Figure 2). Genotypically, efflux pump-associated genes were identified, and the most frequently detected was *smeR*, followed by *smeS*, and the least was *smeD*. Genes conferring resistance to aminoglycosides were detected and these include *aac(6′)-Iak*, *aac(6′)-Iap*, *aac(6′)-Ib3*, *aac(6′)-Iz*, *aph(9)-Ic*, and *aph(3′)-IIc*, with *aph(9)-Ic* and *aph(3′)-IIc* being the most common. Genes conferring resistance to beta-lactams were detected; these were *blaL1*, which was the most frequently detected, and *blaOXA-2* was the least in only one isolate. Finally, the *sul1* gene conferring SXT resistance was detected in only one isolate (Figure 2).

Virulence genes related to adhesion (*smf-1*, *pilT*, *pilQ*, and *gpmA*), biofilm formation (*rmlA* and *spgM*), enzymes (*stmPr1* and *plcN*), and genes related to stress response and survival (*clpP* and *katE*) were detected across all the isolates (100%). BLASTP 2.16.0+ alignment metrics (E-value and Percentage Identity) of protein sequences of the virulence-associated factors of all isolates against the *S. maltophilia* K279a reference genome are illustrated in Supplementary Table S1.

MGEs were detected in (16/21, 76.19%) isolates, with isolate OM-AH-Sm18 showing the highest MGE burden. No MGEs were detected in (5/21, 23.80%) isolates OM-AH-Sm6, OM-AH-Sm7, OM-AH-Sm9, OM-AH-Sm10, and OM-AH-Sm13. Insertion sequences constituted the dominant MGE category, accounting for (37/40, 92.5%) detected elements, whereas composite transposons and unit transposons were less frequent, representing (2/40, 5%) and (1/40, 2.5%) elements, respectively. At the family level, IS110 was the most prevalent insertion sequence family, followed by IS481 and IS3. ISStma6 was the most frequently detected individual element and was predominantly associated with the IS110 family (Table 4).

## 3. Discussion

In this study, most of the Smc isolates (15/20, 75%) were obtained from respiratory tract infections; these samples were mainly sputum and tracheal aspirate samples (7/15, 46.67%), and only one (1/15, 6.67%) bronchoalveolar lavage sample. Bloodstream infections accounted for one isolate only out of the total 20 *S. maltophilia* (1/20, 5%), and wound infections and urinary tract infections only accounted for two isolates each (2/20, 10%). Pourmahdi-Torghabeh et al. [21] also reported the isolation of *S. maltophilia* from different types of infections, such as respiratory infections, urinary tract infections, wound infections, and bloodstream infections, with *S. maltophilia* isolated mostly from the latter. In a twenty-year period, a retrospective study by Song et al. [22], reported that more that 80% of the isolates were from respiratory samples.

Cefiderocol (FDC) is a new siderophore cephalosporin that has recently been approved by the Food and Drug Administration (FDA) for the treatment of certain serious infections caused by a group of Gram-negative bacteria. It is worth mentioning that in vitro data are available; however, clinical studies are still very limited with regard to the treatment of infections caused by *S. maltophilia*, with few studies describing it as a promising, effective treatment [23,24,25].

Traditionally, the backbone of treatment for *S. maltophilia* infections has been SXT [26]; however, this is changing with the updated recommendation by the Infectious Diseases Society of America (IDSA), which recommends the use of SXT as one of at least two-drug combination therapy and orders these antimicrobial agents according to preference, with SXT being the third after FDC and MH [27].

In this study, the findings of antibiotic susceptibility testing show that all Smc isolates (100%) were susceptible to FDC, LEV, and MH. The isolates were least susceptible to TLC, followed by SXT and CL. These findings adhere to and emphasize the importance of the updated IDSA guidelines with regard to management of *S. maltophilia* infections. Similar results regarding FDC susceptibility were also reported by other studies [28,29]. In addition, previous studies reported variable susceptibility patterns to SXT, TLC, LEV, or CL [21,30,31].

Smc members demonstrate an intricate phylogeny due to the conserved 16S rRNA gene sequence [30]. In the present study, the isolates were initially identified using MALDI-TOF MS as belonging to the *S. maltophilia* group. The identification was further confirmed with 16S rRNA sequence analysis and KmerFinder using WGS data. However, upon submission of the WGS of all 21 isolates to NCBI, and using ANI in the checks for submitted genomes, (11/21, 52.38%) of the isolates were confirmed to be *S. maltophilia*, (3/21, 14.29%) isolates were identified as *S. hibiscicola*, and only one isolate (1/21, 4.76%) was identified as *S. pavanii*, one was identified as *S. geniculata*, and one was identified as *S. muris*. However, (4/21, 19.05%) did not match any described type strains within the Smc and were each classified as a distinct *Stenotrophomonas maltophilia* complex sp. OM-AH-Sm3, OM-AH-Sm11, OM-AH-Sm12 and OM-AH-Sm13. To the best of our knowledge, this is the first report of *S. hibiscicola*, *S. pavanii*, and *S. geniculata* from clinical specimens in Oman.

Other studies also reported the identification of *S. hibiscicola*, *S. pavanii*, or *S. geniculata*, and demonstrated that although MALDI-TOF MS is routinely used in clinical laboratories, accurate identification of the closely related species of Smc should be based on ANI or digital DNA–DNA hybridization (dDDH) [30,32].

In the present study, *S. muris* was obtained from the sputum sample of an elderly female patient (OM-AH-Sm15); it showed susceptibility to all antimicrobial agents used in this study. To the best of our knowledge, this is the first time in Oman, and one of the few times in the world, to report *S. muris* from a clinical sample. This is due to the fact that *S. muris* is a recently identified species within the genus, as it was first described in 2022 [33], then its association with human infections (respiratory infection and bloodstream infection) was reported in a study by Liu et al. [34], in 2025, and recently in 2026, a study by Xu et al. [35].

To date, more than 1450 MLST *S. maltophilia* profiles are present on the public databases for molecular typing and microbial genome diversity, pubMLST. In the present study, the most frequently observed ST was ST78 (3/21, 14.29%), and the rest of the isolates (18/21, 85.71%) showed highly diverse STs that were dispersed among the isolates. Moreover, 17 different alleles were identified in 9 different isolates; these isolates formed novel, unique STs, which were subsequently submitted to pubMLST, along with two other isolates that had unique combinations of already known alleles forming new STs. Overall, 12 different STs were identified and submitted to pubMLST. All novel alleles and novel STs were deposited in the pubMLST (https://pubmlst.org/bigsdb?db=pubmlst_smaltophilia_seqdef, accessed on 24 April 2026). Novel alleles of *S. maltophilia* and striking diversity among the STs have been reported previously [14,30,36,37,38,39].

In the present study, already known STs (ST4, ST28, ST31, ST78, ST138 and ST293) were also found, and these were previously reported [14,30,36,37,40].

Smc is well known for its intrinsic resistance to many antimicrobial agents; the presence of acquired resistance mechanisms further limits possible therapeutic options for Smc infections. Intrinsic resistance to aminoglycosides in Smc is mainly due to the chromosomally encoded aminoglycoside-modifying enzymes [27,32].

In the present study, all isolates harbored aminoglycoside-modifying enzymes. The frequency of the genes encoding aminoglycoside resistance ranged from two to three genes per isolate, with (6/21, 28.57%) isolates harboring three genes and most of the isolates (15/21, 71.43%) harboring two genes. The overall detected genes were *aac(6′)-Iak*, *aac(6′)-Iap*, *aac(6′)-Ib3* each detected in (1/21, 4.76%), and *aac(6′)-Iz* in (4/21, 19.05%), *aph(9)-Ic* in (20/21, 95.24%) and *aph(3′)-IIc* in all isolates (100%). It is worth mentioning that there was no striking difference in the distribution of the genes encoding aminoglycoside-modifying enzymes across the different species in the 21 isolates. Interestingly, *aac(6′)-Iap* and *aph(9)-Ic* were recently identified in *S. maltophilia* isolates in a study by Kawauchi et al. [41] and a study by Shi et al. [42], respectively. Additionally, the presence of *aac(6′)-Iak*, *aac(6′)-Ib3 (aacA4)*, *aac(6′)-Iz* and *aph(3′)-IIc* in *S. maltophilia* was previously reported [43].

In this study, metallo-β-lactamase, *blaL1*, was detected in most of the isolates (20/21, 95.24%), as it is a chromosomally encoded β-lactamase [16]. Moreover, *blaOXA-2*, a class D β-lactamase, was found in only one *S. maltophilia* isolate (OM-AH-Sm20) using CARD with perfect matches and using ResFinder with 100% identity in each. It is unusual to report *blaOXA-2* from *S. maltophilia*; however, it is worth mentioning that the isolate that harbored *blaOXA-2* also carried different insertion sequences (IS6100/IS6100R/IS6100L, ISStma2, and ISPa36), which may explain the acquisition of the *blaOXA-2* gene in this isolate.

Trimethoprim/sulfamethoxazole (SXT) has long been used to treat infections caused by Smc, and its resistance is increasingly detected among these isolates. SXT resistance is mediated mainly by *sul* genes [27,30]. The presence of *sul1* harbored by *S. maltophilia* isolates was previously reported [30,32,44,45,46]. Interestingly, although six isolates were phenotypically resistant to SXT, the *sul1* gene was detected in only one isolate. This indicates that SXT resistance in the remaining isolates may be mediated by mechanisms other than *sul1*. Similar findings have been reported in previous studies, in which SXT-resistant *S. maltophilia* isolates did not carry detectable *sul1* genes [38,45,46]. Possible explanations include the involvement of other sulfonamide resistance determinants, mutations affecting the folate biosynthesis pathway, overexpression of multidrug efflux pumps, or additional resistance mechanisms not detected by the ResFinder and CARD databases used in this study. Further phenotypic and functional studies are required to clarify the mechanisms underlying SXT resistance in these isolates.

Smc possesses multidrug efflux pumps, which contribute to the limited activity of antimicrobial agents, including SXT, tetracyclines, and fluoroquinolones [27]. SmeABC is a multidrug efflux pump which belongs to the Resistance-Nodulation-Division (RND) family and confers resistance to beta-lactams, aminoglycosides, and fluoroquinolones [32]. Additionally, SmeDEF is another efflux pump that belongs to the RND family, confers resistance to quinolones and SXT resistance in *S. maltophilia*, and impacts biofilm formation and motility [7,47]. Moreover, SmeRS is the two-component regulatory system for smeABC [32]. In the present study, efflux pumps were detected in (17/21, 80.95%) of the isolates; the efflux pump genes detected are *smeS*, *smeD*, and *smeR*, which belong to the RND family. Other studies have also identified genes belonging to the RND family of efflux pumps [32,40].

*S. maltophilia* harbours different virulence factors, including pili/fimbriae for adhesion and motility, different enzymes such as StmPr1 (serine protease) and PlcN1 (Phospholipase C), factors that help with survival and stress response encoded by genes such as *clpP* genes, and other factors important in biofilm formation, which are encoded by genes such as *rmlA*, and *xanB (spgM)* [7,15,16]. Biofilms are significant to the bacterial ability to resist antibacterial agents as well as immune responses. Also, biofilms contribute significantly to the ability of *S. maltophilia* to survive on different surfaces, including medical equipment [5,7].

In the present study, all the 21 isolates of the different species were found to carry genes contributing to fimbriae and pili formation: *smf-1*, *pilT*, *pilQ*, and *gpmA*, contributing to adhesion. Type 1 fimbriae, Smf-1, is an important virulence factor that contributes to the adherence of *S. maltophilia* to host epithelia, and it plays an important role in the early stages of biofilm formation [7]. While type IV pilus biogenesis protein PilQ is one of the pilus assembly proteins, PilT is one of the twitching motility proteins. GpmA is a glycolytic enzyme phosphoglycerate mutase, which contributes to the adherence of *S. maltophilia* to biotic and abiotic surfaces. Moreover, *gpmA* mutations hinder biofilm formation on both surfaces [48,49].

Studies by Adamek et al. and Kalidasan et al. are among the few studies that investigated the presence of genes related to fimbriae and pili formation among the genomes of *S. maltophilia* isolates K279a (clinical isolate) as well as environmental isolates, and *smf-1*, *pilT*, and *pilQ* were found among all the isolates [50,51]. Different studies reported finding *smf-1* among *S. maltophilia* isolates. Pourmahdi-Torghabeh et al. reported finding *smf-1* in all the isolates [21]. Other studies have reported the presence of *smf-1* in almost all isolates [52,53,54]. Also, this gene has been reported in different species belonging to Smc [30,55]. Moreover, *pilT*, *pilQ*, and *gpmA* have also been previously reported [49,56].

All isolates in this study harboured genes encoding for non-hemolytic phospholipase C (PlcN1) and extracellular serine protease (StmPr1). PlcN1 is implicated in the destruction of lipoprotein membranes, mucin, and immunoglobulins, while StmPr1secretion is mediated via a type II secretion system and is involved in the degradation of fibronectin, fibrinogen, collagen, and interleukin 8 (IL-8) [7,52]. Similarly, Strateva et al. reported finding *plcN1* in 99.1% of tested isolates [52]. However, Saleh et al. reported that *plcN1* was present in 84% of the tested isolates, and Nicolas-Sayago et al. did not find *plcN1* or *stmPr1* in any of the tested isolates [31,57]. Similar to our study, the *stmPr1* gene was reported by Wang et al. in 100% of *S. maltophilia* and not in other species (*S. geniculata*, *S.pavanii* and *S. hibiscicola*), and Fluit et al. reported that *stmPr1* was found in 100% of the *S. maltophilia* isolates and 100% in the other Stenotrophomonas species [30,55]. However, Saleh et al. reported the *stmPr1* gene in 87% of the tested isolates [57].

Moreover, genes encoding stress response (*clpP* and *katE*) were found in all the isolates, belonging to the different species of Stenotrophomonas. ClpP is involved in the processing of defective cytoplasmic proteins and contributes to intrinsic resistance to aminoglycosides. KatE is one of the H_2_O_2_ scavenging enzymes, which act in response to oxidative stress [58,59]. ClpP is a highly conserved protease in prokaryotic cells, and KatE was found to be present in the *S. maltophilia* K279a strain by Crossman et al. [60].

Biofilm formation is the cornerstone enabling persistence of *Stenotrophomonas* infection; all the isolates in this study harboured genes *rmlA* and *xanB (spgM)*, which encode factors important in biofilm formation. These are biofilm-associated genes, one of which is an enzyme with phosphomannomutase and phosphoglucomutase activities (RmlA); a mutation in *rmlAC* was associated with a defective outer membrane layer and impaired biofilm formation [5,61]. Similar to our study, Nicolas-Sayago et al. reported that *rmlA* was present in 100% of isolates [31]. Fluit et al. reported that *rmlA* was present in all of the *S. maltophilia* isolates and 97.8% in the other Stenotrophomonas species [55]. Wang et al. reported the presence of *rmlA* in 36.84% of *S. maltophilia* isolates, and its presence was variable from 0%, 16.67, to 100% in isolates belonging to *S. geniculata*, *S. pavanii*, and *hibiscicola*, respectively [30]. Other studies reported that the presence of *rmlA* was variable among the isolates [21,39,54,57]. XanB, also called SpgM, is a phosphomannose isomerase-GDPmannose pyrophosphorylase, which is involved in the formation of a thicker LPS layer and plays an important role in biofilm formation [5,7,62,63]. In this study, *spgM* was found in all the isolates. This was consistent with a previous study by Bostanghadiri et al., who reported that 100% of the isolates harboured the *spgM* gene [39]. Also, Fluit et al. reported that *spgM* was present in 100% of the *S. maltophilia* isolates and 97.8% in the other Stenotrophomonas species [55]. In addition, other studies reported its variable presence [21,54].

Smc is an opportunistic Gram-negative pathogen notable for its intrinsic and acquired resistance to multiple antibiotics, which is attributed to the presence and activity of mobile genetic elements (MGEs) such as integrons, plasmids, transposons, insertion sequences, and prophages [64,65,66]. These MGEs play a critical role in the horizontal transfer of resistance genes, shaping the genetic diversity and adaptability of *S. maltophilia* in clinical and environmental settings [30,67].

*S. maltophilia* harbors a wide array of MGEs, including plasmids, integrative and conjugative elements (ICEs), integrons, insertion sequences, prophages, and genomic islands [64,65,66].

The present study demonstrated a substantial distribution and diversity of MGEs among clinical Smc isolates, highlighting their potential contribution to genome plasticity and adaptive evolution. MGEs were identified in (16/21, 76.20%) isolates, with isolate (OM-AH-Sm18) exhibiting the highest MGE burden, followed by (OM-AH-Sm20), suggesting increased genomic dynamism and a potentially enhanced capacity for horizontal gene acquisition. In contrast, five isolates lacked detectable MGEs, indicating heterogeneity in MGE carriage among the isolates. Insertion sequences (ISs) represented the predominant MGE category, accounting for (37/40, 92.5%) of all detected elements, whereas composite and unit transposons were comparatively uncommon. The predominance of IS elements is consistent with previous studies demonstrating that ISs constitute a major mobilome component in *S. maltophilia* and contribute to genomic rearrangements, gene disruption, and mobilization of adaptive determinants [64,66].

In our study, among the detected IS families, IS110 was the most prevalent, followed by IS481 and IS3, suggesting lineage-specific enrichment of particular transposition systems within the studied isolates. Notably, ISStma6 was the most predominantly associated with the IS110 family and the most frequently identified element, indicating possible selective maintenance or expansion of this element in *S. maltophilia* genomes. The marked predominance of ISs may, therefore, reflect ongoing microevolutionary processes driven by selective pressures within clinical environments, including antimicrobial exposure and host-associated stress conditions [30,64,66]. Large-scale comparative genomics further reveals that the *S. maltophilia* complex is highly diverse, with lineage-specific enrichment of MGEs such as ICEs, integrons, transposases, and prophages, which correlate with source and geographic distribution. Furthermore, the observed variability in MGE content among isolates supports previous reports describing substantial genomic heterogeneity and lineage-dependent mobilome profiles in *S. maltophilia* populations [30,64,68].

## 4. Materials and Methods

### 4.1. Study Design and Sample Collection

This study is a prospective cross-sectional study conducted in Sultan Qaboos University (SQU) in collaboration with Sultan Qaboos University Hospital (SQUH). A total of 21 clinical isolates of Smc were collected (with clinical deduplication to 20 unique episodes detailed in the Section 2, Table 1) from different samples, respiratory, blood culture, wound swab, and urine, submitted to the Microbiology Diagnostic Laboratory in SQUH, during the period from the start of March 2025 to the end of August 2025. The clinical isolates of Smc were identified as belonging to the *S. maltophilia* group, using MALDI-TOF MS (Bruker, Munich, Germany), using the Bruker Daltonics Applications Library (BDAL, Revision 13), the synchronized In Vitro Diagnostics clinical library (IVD_equivalent, Revision 13), and the specialized Bruker Filamentous Fungi Library (Revision 7). Ethical approval was obtained from the Medical Research Ethics Committee (MREC), College of Medicine & Health Sciences, SQU (REF. NO. SQU-EC/280\2024).

The collected Smc clinical isolates were preserved at −80 °C in the CRYOBANK bacterial preservation and storage system (Mast Group Ltd., Bootle, UK). For downstream processing, these isolates were subcultured on sheep blood agar (Oxoid, Basingstoke, UK).

### 4.2. Antibiotic Susceptibility Testing

Antimicrobial susceptibility testing was performed using the disc diffusion method for trimethoprim/sulfamethoxazole (SXT, 25 μg), minocycline (MH, 30 μg), levofloxacin (LEV, 5 μg), and cefiderocol (FDC, 30 μg), while the E-test was used for ticarcillin/clavulanic acid (TLC) and chloramphenicol (CL). Muller–Hinton agar was used in susceptibility testing. Muller–Hinton agar and the antibiotic discs were purchased from (Oxoid, Basingstoke, UK). E-tests were purchased from (BioMérieux, Marcy-l’Étoile, France). The tests were conducted, and the results were interpreted according to CLSI 2025 [20].

### 4.3. DNA Extraction and Whole Genome Sequencing

Whole genome sequencing (WGS) was carried out for the 21 isolates identified as *S. maltophilia*. First, DNA extraction was done using QIAamp DNA Mini Kit and an EDTA-free EB buffer (Qiagen, Hilden, Germany) on an overnight bacterial culture in Nutrient broth (Oxoid, Basingstoke, UK). The purity of the extracted DNA was assessed using Nanodrop (IMPLEN/nanophotometer N120, Munich, Germany). The concentration of the extracted DNA was then quantified using Qubit (Thermo Fisher Scientific, Waltham, MA, USA), and the integrity of the extracted genomic DNA was checked against GeneRuler 1 kb DNA ladder (Thermo Fisher Scientific, Waltham, MA, USA) using 1% agarose gel electrophoresis (ACROS Organics agarose LE, Thermo Fisher Scientific, Waltham, MA, USA). Electrophoresis was performed at 100 V for 30 min, and the bands were visualized using Invitrogen iBright1500 (Thermo Fisher Scientific, Waltham, MA, USA).

Then, WGS was carried out using the Illumina platform MicrobesNG (https://microbesng.co.uk, Birmingham, UK, accessed on 15 April 2026).

DNA libraries were prepared using standard Illumina library preparation methods, then sequencing was performed on an Illumina NextSeq platform to give paired-end reads, followed by quality control assessment and trimming. Raw reads were de novo assembled using SPAdes (version 3.7) with a minimum coverage of 60×. The resulting contigs were then annotated using Prokka (version 1.11) [69,70].

The assembled genomes were then analysed using different online tools; pubMLST database (University of Oxford, Oxford, UK) (https://pubmlst.org/bigsdb?db=pubmlst_smaltophilia_seqdef&l=1&page=sequenceQuery, accessed on 24 April 2026) to identify the different sequence types (ST) [71]. MGEfinder in the Center for Genomic Epidemiology (CGE, Lyngby, Denmark) (https://cge.food.dtu.dk/services/MobileElementFinder/, accessed on 7 April 2026) was used to detect mobile genetic elements (MGEs) [72]. ResFinder version 4.7.2 in CGE (Lyngby, Denmark) (https://genepi.food.dtu.dk/resfinder, accessed on 5 April 2026) [73] and the Comprehensive Antibiotic Resistance Database (CARD) version 4.0.1, with (McMaster University, Hamilton, Canada) (https://card.mcmaster.ca/analyze/rgi, accessed on 1 April 2026) [74] were used to identify different genes associated with antimicrobial resistance. The results of ResFinder and the strict and perfect results of CARD that fulfill an equal or above 80% identity of matching region and equal or above 60% length of reference sequence were reported.

To identify different virulence genes, the annotated Genbank files resulting from the WGS were then used to identify genes associated with adhesion (*smf-1*, *pilT*, *pilQ*, and *gpmA*), biofilm formation (*rmlA* and *spgM*), enzymes (*stmPr1* and *plcN*), and genes related to stress response and survival (*clpP* and *katE*). Then, the presence of these genes was further validated by performing a BLASTP 2.16.0+ [75,76] of the amino acids in the Genbank files search against the UniProt database [77] (UniProt Consortium; Hinxton, UK/Geneva, Switzerland/Washington, D.C., USA) (https://www.uniprot.org/blast, accessed on 18 April 2026). Criteria for confirmation included a sequence identity >70% relative to the *S. maltophilia* reference strain K279a, and E-values of zero (or very low E-values approaching zero) were reported.

CSI Phylogeny in CGE (https://cge.food.dtu.dk/services/CSIPhylogeny/, accessed on 31 March 2026) [78] was used to construct the phylogenetic tree, which was then visualized and annotated using Interactive Tree of Life (iTOL, version 7) (https://itol.embl.de/, accessed on 17 May 2026) [79].

KmerFinder 3.2 in GCE was used to confirm the identification of the isolates to the species level (https://cge.food.dtu.dk/services/KmerFinder/, accessed on 30 April 2026) [80,81,82]. The correct identification to species level was done by the National Center for Biotechnology Information (NCBI) on submission of the WGS data using average nucleotide identity (ANI) against the genomes of the type strains already present in GenBank [83,84].

This Whole Genome Shotgun project has been deposited at DDBJ/ENA/GenBank under the accession numbers: JBXVPA000000000 to JBXVPI000000000, JBXVOX000000000 to JBXVOZ000000000, and JBYBLI000000000 to JBYBLQ000000000. The details are shown in Supplementary Table S2.

## 5. Conclusions

This study showed high ST diversity among the studied isolates, with newly identified alleles and STs. Among the isolates, *S. muris*, *S. geniculata*, *S. pavanii*, and *S. hibiscicola* were identified using ANI during NCBI checks, which are reported for the first time in Oman from clinical isolates. These isolates showed no specific pattern in terms of antibiotic susceptibility; all isolates were found to be susceptible to FDC, MH, and LEV. Moreover, virulence-associated genes (*smf-1*, *pilT*, *pilQ*, *gpmA*, *rmlA*, *spgM*, *stmPr1*, *plcN*, *clpP*, and *katE*) were highly conserved across all isolates. Furthermore, resistance genes (*blaOXA-2*, *blaL1*, and *sul1*) demonstrated variable distribution patterns among isolates. More studies are needed to establish relationships between the different members of the Smc and the different molecular resistome and virulome.

## 6. Limitations

This study has several limitations. First, it was conducted at a single tertiary care hospital, SQH, and included 21 Smc clinical isolates collected over a six-month period. Therefore, the findings may not fully represent the broader epidemiology, genomic diversity, or antimicrobial resistance patterns of the Smc across Oman. Nevertheless, despite the limited sample size, the study identified novel alleles, new sequence types, and a Stenotrophomonas species reported from Oman for the first time, highlighting the value of genomic surveillance even in small preliminary datasets. Second, although basic clinical metadata were included in this study, detailed clinical information and patient outcomes were not comprehensively available. Third, the genomic interpretation of Smc isolates remains challenging due to the complexity of taxonomy and species-level identification within the complex, as well as the lack of dedicated and comprehensive virulence factor databases specific to this organism.

## Figures and Tables

**Table 1:** Characteristics of the studied Smc isolates (*n* = 20).

Isolates	Type of Sample	Ward	Gender	Age
OM-AH-Sm1	Tracheal Aspirate	ICU	M	73 years
OM-AH-Sm2 *	Tracheal Aspirate	PICU	F	<1 year
OM-AH-Sm3 **	Wound Swab	PICU	F	<1 year
OM-AH-Sm5	Central line	Female medical	F	32 years
OM-AH-Sm6	Tracheal Aspirate	High dependency	F	45 years
OM-AH-Sm7	Tracheal Aspirate	PICU	M	<1 year
OM-AH-Sm8	Sputum	High dependency (Pediatric)	M	13 years
OM-AH-Sm9	Sputum	Hematology	F	35 years
OM-AH-Sm10	Sputum	High dependency	M	59 years
OM-AH-Sm11	Mid-stream urine	Surgical	F	40 years
OM-AH-Sm12	Sputum	Pediatric	M	10 years
OM-AH-Sm13	Sputum	NICU	F	<1 year
OM-AH-Sm14	Wound Swab	ICU	M	68 years
OM-AH-Sm15	Sputum	Female medical	F	85 years
OM-AH-Sm16	Tracheal aspirate	ICU	M	40 years
OM-AH-Sm17	Bronchoalveolar lavage	Pediatric hematoncology	M	<1 year
OM-AH-Sm18	Mid-stream urine	Hematology	M	71 years
OM-AH-Sm19	Tracheal aspirate	PICU	M	<1 year
OM-AH-Sm20	Sputum	General pediatric	F	1 year
OM-AH-Sm21	Tracheal aspirate	ICU	F	63 years

ICU: Intensive care unit; PICU: Pediatric Intensive care unit; NICU: Neonatal Intensive care unit. * A total of 21 consecutive clinical isolates were collected, sequenced, and deposited in the NCBI database. To prevent patient-repetition bias, this dataset was deduplicated to 20 unique isolates for downstream demographic and phenotypic susceptibility profiling. OM-AH-Sm4 was excluded from downstream patient demographic profiling and phenotypic susceptibility metrics because it is an isolate from a sputum sample collected on Day 10 from a patient already represented by a Day 1 tracheal aspirate sample (OM-AH-Sm2). ** Isolate (OM-AH-Sm3) was recovered from a distinct sample type (wound swab) from the same patient (who provided OM-AH-Sm2); it remains in the analysis as it represents a distinct clinical episode.

**Table 2:** MIC values for CL and TLC using E-test for the Smc isolates (*n* = 20).

	CL (MIC µg/mL)	S/I/R ^1^	TLC (MIC µg/mL)	S/I/R ^1^
Om-AH-Sm1	2	S	32	I
Om-AH-Sm2 *	2	S	1	S
Om-AH-Sm3	8	S	>256	R
Om-AH-Sm5	12	I	24	I
Om-AH-Sm6	8	S	12	S
Om-AH-Sm7	12	I	48	I
Om-AH-Sm8	6	S	3	S
Om-AH-Sm9	8	S	>256	R
Om-AH-Sm10	6	S	3	S
Om-AH-Sm11	4	S	32	I
Om-AH-Sm12	16	I	24	I
Om-AH-Sm13	8	S	48	I
Om-AH-Sm14	16	I	>256	R
Om-AH-Sm15	2	S	16	S
Om-AH-Sm16	4	S	1.5	S
Om-AH-Sm17	0.75	S	48	I
Om-AH-Sm18	6	S	24	I
Om-AH-Sm19	1.2	S	48	I
Om-AH-Sm20	4	S	6	S
Om-AH-Sm21	2	S	2	S

^1^ S/I/R were interpreted according to CLSI 2025 [20]. * OM-AH-Sm4, (collected on Day 10 tracheal aspirate, which was excluded from population percentages) exhibited the following distinct MIC values: CL, 2 µg/mL (S); TLC, 1 µg/mL (S).

**Table 3:** Distribution of different alleles, including the newly introduced alleles and the new STs, among the 21 isolates. New alleles and STs are shown in bold.

Isolate	Alleles							Sequence Types
	*atpD*	*gapA*	*guaA*	*mutM*	*nuoD*	*ppsA*	*recA*	
**Om-AH-Sm1**	3	4	24	7	7	22	7	31
**Om-AH-Sm2**	1	4	18	46	8	70	58	78
**Om-AH-Sm3**	13	**393**	**911**	216	72	143	22	**1443**
**Om-AH-Sm4**	1	4	18	46	8	70	58	78
**Om-AH-Sm5**	103	89	**912**	73	72	239	135	**1445**
**Om-AH-Sm6**	13	223	805	**416**	89	144	198	**1446**
**Om-AH-Sm7**	156	8	**913**	185	4	**426**	308	**1447**
**Om-AH-Sm8**	4	22	82	5	70	6	1	138
**Om-AH-Sm9**	78	136	**914**	306	72	**427**	135	**1448**
**Om-AH-Sm10**	**295**	**394**	**915**	228	173	**428**	**374**	**1449**
**Om-AH-Sm11**	72	77	486	166	71	**429**	67	**1450**
**Om-AH-Sm12**	95	77	**916**	60	71	85	202	**1451**
**Om-AH-Sm13**	72	77	458	86	172	85	202	**1452**
**Om-AH-Sm14**	**296**	171	**917**	227	72	251	79	**1453**
**Om-AH-Sm15**	2	134	214	45	73	69	128	293
**Om-AH-Sm16**	1	4	7	7	28	19	6	4
**Om-AH-Sm17**	4	3	2	5	9	6	9	28
**Om-AH-Sm18**	82	250	434	187	66	283	178	**1454**
**Om-AH-Sm19**	3	373	24	7	1	1	7	1417
**Om-AH-Sm20**	16	**395**	**918**	88	69	375	72	**1455**
**Om-AH-Sm21**	1	4	18	46	8	70	58	78

**Table 4:** Distribution of mobile genetic elements (MGEs) among the Smc isolates.

Isolate	Total MGE Copies	No. of Insertion Sequences	No. of Composite Transposons	No. of Unit Transposons	Detected MGEs	Family
Om-AH-Sm1	3	3	0	0	ISStma1, ISStma6, ISStma7	IS481 IS110 IS110
Om-AH-Sm2	2	2	0	0	ISStma6, ISStma7	IS110 IS110
Om-AH-Sm3	2	2	0	0	ISStma7, ISStma12	IS110 IS481
Om-AH-Sm4	3	3	0	0	ISStma1, ISStma6, ISStma7	IS481 IS110 IS110
Om-AH-Sm5	1	1	0	0	ISStma2	IS3
Om-AH-Sm6	0	0	0	0	-	-
Om-AH-Sm7	0	0	0	0	-	-
Om-AH-Sm8	4	4	0	0	ISStma1, ISStma6, ISStma7, ISStma12	IS481 IS110 IS110 IS481
Om-AH-Sm9	0	0	0	0	-	-
Om-AH-Sm10	0	0	0	0	-	-
Om-AH-Sm11	2	1	0	1	ISStma2, Tn501	IS3 Tn3
Om-AH-Sm12	1	1	0	0	ISStma2	IS3
Om-AH-Sm13	0	0	0	0	-	-
Om-AH-Sm14	1	1	0	0	ISStma4	IS110
Om-AH-Sm15	1	1	0	0	ISStma6	IS110
Om-AH-Sm16	1	1	0	0	ISStma1	IS481
Om-AH-Sm17	1	1	0	0	ISStma6	IS110
Om-AH-Sm18	7	5	2	0	ISStma6 *, cn_13529_ISStma6, cn_16307_ISStma6	IS110
Om-AH-Sm19	3	3	0	0	ISStma6 ISStma7 ISStma14	IS110 IS110 IS3
Om-AH-Sm20	5	5	0	0	IS6100/ IS6100R/IS6100L ISStma2 ** ISPa36	IS6 IS3 IS21
Om-AH-Sm21	3	3	0	0	ISStma1 ISStma6 ISStma7	IS481 IS110 IS110
Total	40	37	2	1		

* The frequency is 5; ** the frequency is 3.

## Data Availability

The original data for this study are available within the manuscript, and any additional inquiries can be sent to the corresponding author.

## Supplementary Materials

The following supporting information can be downloaded at https://www.mdpi.com/article/10.3390/antibiotics15060600/s1: Supplementary Table S1: BLASTp Alignment Metrics (E-value and Percentage Identity) of *Stenotrophomonas maltophilia* Complex Protein Sequences of certain virulence associated factors Against the *S. maltophilia* K279a Reference Genome; Supplementary Table S2: shows the accession numbers under which the Whole Genome Shotgun project has been deposited at DDBJ/ENA/GenBank. The BioProject ID is PRJNA1456569.

## Abbreviations

The following abbreviations are used in this manuscript:

Smc	*Stenotrophomonas maltophilia* complex
SQU	Sultan Qaboos University
SQUH	Sultan Qaboos University Hospital
MREC	Medical Research Ethics Committee
SXT	Trimethoprim/sulfamethoxazole
MH	Minocycline
LEV	Levofloxacin
FDC	Cefiderocol
TLC	Ticarcillin/clavulanic acid
CL	Chloramphenicol
CLSI	Clinical and Laboratory Standards Institute
WGS	Whole genome sequencing
MGEs	Mobile genetic elements
CARD	Comprehensive Antibiotic Resistance Database
NCBI	National Center for Biotechnology Information
ANI	Average nucleotide identity
dDDH	Digital DNA–DNA hybridization
ISs	Insertion sequences
MLST	Multilocus Sequence Typing
