# Supplementary material for: Stenotrophomonas maltophilia Complex: Genomic Characterization, Antimicrobial Resistance and First Report of S. muris from Oman
Source: Antibiotics (Basel). 2026 Jun 12;15(6):600. doi: 10.3390/antibiotics15060600 (PMC13296070; doi:10.3390/antibiotics15060600)
Supplement: Supplementary file 1 [file antibiotics-15-00600-s001.zip › Supplementary Table S1.pdf]

Supplementary Table S1: BLASTp Alignment Metrics (E-value and Percentage Identity) of *Stenotrophomonas maltophilia* Complex Protein Sequences of certain virulence associated factors Against the *S. maltophilia* K279a Reference Genome

Isolates	Smf-1		PilT		PilQ		GpmA		PlcN1		StmPr1		ClpP		KatE		SpgM		RmlA	
	%Identity*	E-value*	%Identity*	E-value*	%Identity*	E-value*	%Identity*	E-value*	%Identity*	E-value*	%Identity*	E-value*	%Identity*	E-value*	%Identity*	E-value*	%Identity*	E-value*	%Identity*	E-value*
OM-AH-Sm1	100	3.90E-116	100	0	100	0	99.6	0	97.9	0	100	0	100	1.10E-143	99.9	0	100	0	90.8	0
OM-AH-Sm2	100	3.90E-116	100	0	100	0	99.6	0	99	0	100	0	100	1.10E-143	99.7	0	98.9	0	100	0
OM-AH-Sm3	97.6	2.20E-113	100	0	92.4	0	98.4	1.90E-180	91.9	0	97.2	0	100	1.10E-143	96.2	0	96.9	0	94.6	0
OM-AH-Sm4	100	3.90E-116	100	0	100	0	99.6	0	99	0	100	0	100	1.10E-143	99.7	0	98.9	0	100	0
OM-AH-Sm5	97.6	2.20E-113	99.7	0	91.8	0	98	4.60E-179	94.3	0	95.2	0	99.5	1.50E-143	97.1	0	97.1	0	97.3	0
OM-AH-Sm6	97.6	2.20E-113	100	0	93.3	0	99.2	0	91.9	0	97.6	0	100	1.10E-143	96.7	0	96.2	0	94.9	0
OM-AH-Sm7	93.5	4.70E-108	99.7	0	91.6	0	98.4	1.10E-179	93.6	0	95.9	0	100	1.10E-143	96.7	0	95.5	0	94.9	0
OM-AH-Sm8	97.6	1.20E-112	100	0	100	0	99.2	0	99.9	0	100	0	100	1.10E-143	99.6	0	98.7	0	94.9	0
OM-AH-Sm9	97.6	2.20E-113	99.7	0	91.5	0	99.2	0	92.5	0	95.5	0	99.5	1.50E-143	97	0	96.9	0	97.6	0
OM-AH-Sm10	93.5	1.10E-108	98.8	0	89.7	0	98.8	0	85.6	0	95.9	0	98.1	7.30E-142	90.7	0	73.7	0	92.9	0
OM-AH-Sm11	97.6	2.20E-113	100	0	92.4	0	98.8	0	91.9	0	96.6	0	100	1.10E-143	97.1	0	96.9	0	93.6	0
OM-AH-Sm12	97.6	2.20E-113	100	0	92.5	0	98.8	0	91.7	0	96.6	0	100	1.10E-143	97	0	96.7	0	96.6	0
OM-AH-Sm13	97.6	2.20E-113	100	0	93.1	0	98.8	0	92	0	97.6	0	100	1.10E-143	97	0	96.9	0	96.6	0
OM-AH-Sm14	97.6	2.20E-113	99.7	0	91.8	0	99.2	0	97.6	0	94.5	0	99.5	1.50E-143	99	0	96.9	0	96.9	0
OM-AH-Sm15	97.6	2.20E-113	100	0	92.4	0	99.2	0	93.2	0	96.9	0	99.5	1.50E-143	97.6	0	94.4	0	93.2	0
OM-AH-Sm16	100	3.90E-116	100	0	100	0	99.6	0	98.3	0	99.7	0	99.5	4.40E-143	99.9	0	98.9	0	100	0
OM-AH-Sm17	97.6	1.20E-112	100	0	99.8	0	99.2	0	96	0	100	0	100	1.10E-143	99.6	0	98.7	0	95.3	0
OM-AH-Sm18	97.6	2.20E-113	100	0	92.5	0	98.8	0	88.1	0	96.6	0	99.5	1.50E-143	96.4	0	94.6	0	95.6	0
OM-AH-Sm19	100	3.90E-116	100	0	99.8	0	99.2	0	97.7	0	99.8	0	100	1.10E-143	99.9	0	100	0	90.8	0
OM-AH-Sm20	94.7	2.00E-109	99.7	0	91.8	0	98	4.60E-179	92.6	0	96.2	0	100	1.10E-143	97.4	0	97.3	0	96.3	0
OM-AH-Sm21	100	3.90E-116	100	0	99.8	0	99.6	0	99	0	100	0	100	1.10E-143	99.7	0	98.9	0	100	0

*Compared to Stenotrophomonas maltophilia (strain K279a)
