# Supplementary material for: Stenotrophomonas maltophilia Complex: Genomic Characterization, Antimicrobial Resistance and First Report of S. muris from Oman
Source: Antibiotics (Basel). 2026 Jun 12;15(6):600. doi: 10.3390/antibiotics15060600 (PMC13296070; doi:10.3390/antibiotics15060600)
Supplement: Supplementary file 1 [file antibiotics-15-00600-s001.zip › Supplementary Table S2.pdf]

Supplementary Table S2: shows the accession numbers under which the Whole Genome Shotgun project has been deposited at DDBJ/ENA/GenBank. The BioProject ID is PRJNA1456569.

**Isolates**	**Accession Number**
Om-AH-Sm1	JBXVPI000000000
Om-AH-Sm2	JBXVPH000000000
Om-AH-Sm3	JBYBLQ000000000
Om-AH-Sm4	JBXVPG000000000
Om-AH-Sm5	JBYBLP000000000
Om-AH-Sm6	JBXVPF000000000
Om-AH-Sm7	JBYBLO000000000
Om-AH-Sm8	JBXVPE000000000
Om-AH-Sm9	JBYBLN000000000
Om-AH-Sm10	JBXVPD000000000
Om-AH-Sm11	JBYBLM000000000
Om-AH-Sm12	JBYBLL000000000
Om-AH-Sm13	JBYBLK000000000
Om-AH-Sm14	JBYBLJ000000000
Om-AH-Sm15	JBXVPC000000000
Om-AH-Sm16	JBXVPB000000000
Om-AH-Sm17	JBXVPA000000000
Om-AH-Sm18	JBYBLI000000000
Om-AH-Sm19	JBXVOZ000000000
Om-AH-Sm20	JBXVOY000000000
Om-AH-Sm21	JBXVOX000000000
